# MMP7 Is Required to Mediate Cell Invasion and Tumor Formation upon Plakophilin3 Loss

**DOI:** 10.1371/journal.pone.0123979

**Published:** 2015-04-13

**Authors:** Srikanta Basu, Rahul Thorat, Sorab N. Dalal

**Affiliations:** Advanced Centre for Treatment Research and Education in Cancer (ACTREC), Tata Memorial Centre, Kharghar Node, Navi Mumbai, Maharashtra, India; Sun Yat-sen University Medical School, CHINA

## Abstract

Plakophilin3 (PKP3) loss results in increased transformation in multiple cell lines in vitro and increased tumor formation in vivo. A microarray analysis performed in the PKP3 knockdown clones, identified an inflammation associated gene signature in cell lines derived from stratified epithelia as opposed to cell lines derived from simple epithelia. However, in contrast to the inflammation associated gene signature, the expression of MMP7 was increased upon PKP3 knockdown in all the cell lines tested. Using vector driven RNA interference, it was demonstrated that MMP7 was required for in-vitro cell migration and invasion and tumor formation in vivo. The increase in MMP7 levels was due to the increase in levels of the Phosphatase of Regenerating Liver3 (PRL3), which is observed upon PKP3 loss. The results suggest that MMP7 over-expression may be one of the mechanisms by which PKP3 loss leads to increased cell invasion and tumor formation.

## Introduction

Matrilysin (MMP7) is one of the smallest members of the MMP family and is a highly potent metallo-protease which can degrade casein, laminin, fibronectin, collagen III/IV/V/IX/X/XI, type I/II/IV/ V gelatins, elastin and proteoglycans [1, 2]. MMP-7 is secreted specifically by epithelial cells [3] and its over-expression has been observed in many tumor types such as colorectal cancer [4–7], epidermolysisbullosa associated skin cancer [8, 9], bladder cancer [10], gastric cancers [3, 11], pancreatic cancer [12] and esophageal cancer [13, 14]. An increase in the levels of MMP-7 mRNA was observed to correlate with increased dedifferentiation and metastasis in colon cancers [5, 15]. Loss of MMP7 either by antisense RNA mediated knockdown in colorectal cancer cell lines or by knockout in mice leads to decreased tumor incidence, while an increase in MMP7 expression causes increased tumor formation [16–18].

Desmosomes are cell-cell adhesion junctions present in both simple and stratified epithelial cells. Desmosomes anchor intermediate filaments in adjoining cells and thus play a central role in the formation of a tissue wide intermediate filament network, allowing cells to survive when they encounter mechanical stress during tissue repair [19, 20]. Three major protein families contribute to desmosome assembly, the desmosomal cadherins (desmogleins and desmocollins), the plakin family (desmosplakin) and the ARM repeat containing proteins (plakoglobin and plakophilins) (reviewed in [19, 20]).

Plakophilin3 (PKP3) is the most widely expressed plakophilin family member and is ubiquitously present in all the layers of the stratified epithelia and simple epithelia except in hepatocytes [21]. PKP3 forms a complex with a broad repertoire of desmosomal proteins like the desmosomal cadherins like desmoglein 1–3, desmocollins 1 and 3; keratin 18; desmoplakin and plakoglobin [22]. PKP3 plays a crucial role in the maintenance of the desmosomal structure and function by mediating recruitment of other desmosomal components to the cell border [23]. Previous results from our laboratory demonstrated that PKP3 loss leads to alterations in desmosome size, a decrease in cell-cell adhesion, increased cell migration and an increase in colony formation in soft agar and tumor formation and metastasis in immune-compromised mice [24]. PKP3 expression is known to decrease in high grade poorly differentiated oropharyngeal cancer [25], colon cancer [26], gastric cancer [27] and bladder cancers [28]. The epidermis of PKP3 knock-out mice (PKP3-/-) show increase in epidermal proliferation, hair loss and are more prone to cutaneous inflammation. Under normal conditions, young PKP3-/- mice, of the age of 3 weeks develop epidermal hyperplasia, severe skin inflammation and hair loss. In older mice, the inflammation persists and is accompanied by enlargement of regional lymph nodes [29].

Recent results from our lab have shown that PKP3 loss leads to an increase in PRL3 (Phosphatase of regenerating liver-3) protein levels leading to the dephosphorylation of keratin8 (K8), which results in increased neoplastic progression and metastasis [30]. To determine if alterations in the expression of other gene products were observed upon PKP3 knockdown, an expression analysis was performed comparing vector control cells to PKP3 knockdown cells. PKP3 knockdown clones generated from cell lines derived from the stratified epithelia (HaCaT and FBM) show an increase in expression of many inflammation associated genes and these changes were not observed in PKP3 knockdown clones derived from HCT116 cells. However, in contrast to other gene products, MMP7 mRNA and protein levels were increased upon PKP3 loss, in all cell lines tested. Our results suggest that in HCT116 cells, the increase in MMP7 levels is driven by PRL-3 over-expression in the PKP3 knockdown clones and that MMP7 is required for tumor formation in-vivo upon PKP3 loss.

## Materials and Methods

### Plasmids and constructs

The oligonucleotides used to generate the MMP7 shRNA constructs (S1 Table) were cloned downstream of the U6 promoter in pLKO.1-EGFPf-puro [31] digested with AgeI and EcoRI.

### Cell culture and transfections

HCT116, HaCaT and FBM cells (Fetal buccal mucosal cell line) were cultured as described previously [23, 24, 30]. HCT116, HaCaT derived PKP3 knockdown clones and the respective vector control clones were maintained in selection media containing 5μg/ml of blasticidin, while the FBM derived PKP3 knockdown and the vector control clones were maintained in media containing 0.5μg/ml puromycin as described [24]. To generate HCT116 derived stable double knockdown clones for PKP3 and MMP7, the PKP3 knockdown clone, shpkp3-2 was transfected with MMP7 targeted shRNA encoding plKO.EGFP-f plasmid using Lipofectamine LTX reagent (Life Technologies). The cells were maintained in media containing 5μg/ml blasticidin and 0.5μg/ml puromycin to obtain single cell clones. The PRL3 inhibitor-1 (Sigma) was added to cells in culture at a concentration of 10μM for either 24 or 48 hours.

### Microarray analysis

RNA isolated from the FBM derived vector control and PKP3 knockdown cells and HCT116 derived vector control and PKP3 knockdown cells were Cy3 labeled and processed for the Sureprint G3 Human GE 8x60k microarray by single color hybridization. The results obtained from the microarray were analyzed using the Agilent Feature Extraction software. Using normalized signal intensities (g-processed signal) obtained from the microarray, the fold changes of genes altered in FBM derived PKP3 knockdown clone (shpkp3-2) has been compared to the vector control clone (vec). Similarly, the HCT116 derived derived PKP3 knockdown clone (shpkp3-2) has been compared to the vector control clone (vec). A functional classification of the differentially regulated genes was performed using GeneSpring GX 11.0 software and gene ontology browser. The significant pathway list for differentially regulated genes was obtained using the GeneSpring GX 11.0 and Biointerpreter software (Genotypic, Bangalore, India). The data for the FBM derived clones has been deposited in the NCBI GEO database (Accession number GSE61512), while the data for the HCT116 derived clones have been deposited in the NCBI GEO database (Accession number GSE64580). Functional classification of genes altered upon PKP3 loss was performed using the PANTHER Classification System software [32, 33].

### Isolation of total RNA, real time PCR reactions and reverse transcriptase coupled PCR reactions

The forward and reverse oligonucleotides used in this study are shown in S1 Table. Cells were collected in RLT buffer and total RNA isolated using the Qiagen RNeasy Kit, following the manufacturer’s protocol and 2μg of RNA was reverse transcribed to cDNA using the ABI High Capacity Reverse Transcriptase Kit (Applied Biosystems, Life Technologies). The cDNA obtained was used for SyBr Green based Real time PCR using ABI SyBr Green PCR Master mix (Applied Biosystems, Life Technologies). Real Time PCR was performed using 10ng cDNA per reaction using the QuantStudio 12K Flex Real-Time PCR System (Life Technologies). The fold change in relative expression of each target gene compared to the loading control was calculated using the 2^(-ΔΔCt)^ method [34]. A change in expression of two-fold either way was considered significant. For reverse transcriptase coupled polymerase chain reactions, each reaction contained either 500ng or 1μg cDNA the fragments were amplified using Taq DNA polymerase (New England Biolabs).

### Antibodies and Western blot analysis

For Western blots, the mouse monoclonal β actin antibody (clone AC74, catalogue number A5316, Sigma) was used at a dilution of 1:5000, the mouse monoclonal PKP3 antibody (clone 23E34, catalogue number 35–7600, Invitrogen) at a dilution of 1:2000, mouse monoclonal MMP7 antibody (clone JL07, sc-80205, Santa Cruz,) at a dilution of 1:100. Goat anti-mouse secondary antibody (Pierce) was used at a dilution of 1:2500. The blots were developed using Supersignal West Pico Cheminiluminescent Substrate (Pierce) according to the manufacturer’s instructions. Cells were lysed in 1X sample buffer as described [24] and protein concentration quantitated using Folin-Lowry’s method. 75 μg of the extract was resolved on 10% SDS-PAGE gels and transferred to nitrocellulose membranes (Mdi, Membrane Technologies) followed by Western blotting with the indicated antibodies. Western blots for MMP7 were performed as follows. Cells growing in serum containing media were replaced with fresh media and incubated for 24 hours or 48 hrs with or without the PRL3 inhibitor and the cell culture supernatants centrifuged for 10 mins at 7500xg to remove any cell debris. Three volumes of acetone were added to the supernatant and the reaction incubated at -20°C for 24 hours and then centrifuged at 4500xg for 15 minutes at 4°C. The precipitate obtained was washed twice with acetone and the pellet air dried at RT for 16 hours. The precipitate was boiled in 1X SDS lysis buffer (2% SDS, 50mM Tris pH 6.8), then diluted ten-fold in 1X SDS lysis buffer and the protein concentration was measured using Folin-Lowry’s method. 100μg of the lysate was resolved on 12% SDS-PAGE gels and transferred to nitrocellulose followed by Western blots with antibodies to MMP7. The blot was stained with Ponceau-S (Sigma) to demonstrate equal loading.

### Scratch wound healing assays and matrigel invasion assays

Scratch wound healing assays were performed as described [30]. The plates were visualized under an Axiovert 200M Inverted microscope (Carl Zeiss) fitted with a cell incubator stage maintained at 37°C and 5% CO_2_. Cells were observed by time lapse microscopy and images taken every 10 minutes for 20 hours using the AxioCamMRm Camera (Zeiss) with a 10X phase I objective. Axiovision software version 4.8 (Ziess) was used to measure cell migration. Three independent experiments were performed in triplicates for each clone. Invasion assays were performed as described [35, 36]. Briefly, 2x10^5^ cells resuspended in 200μl of serum free media were added to the upper chambers and 400μl of serum containing media was added in the lower chamber. The inner side of the insert was pre-coated with 5μg of Matrigel (BD Biosciences). After 24 hours, cell culture inserts were then removed from the wells and the cells attached to the inner side of the insert were removed using cotton buds. The inserts with cells on the outer side of the membrane were fixed with 4% para-formaldehyde, stained with 1% crystal violet and mounted on slides using DPX mountant (Qualigens). Images were taken using Olympus SZ61 stereo microscope using a 10X objective lens. Three independent experiments were performed for each clone.

### Soft agar assays

2500 cells of the HCT116 based plakophilin3 knockdown clones shpkp3-1 and shpkp3-2, the vector control clone (vec), the shpkp3-2 derived vector control and shpkp3-2 derived double knockdown clones were resuspended in 0.4% soft agarose as described [24]. The cells were maintained in media containing blasticidin (for vec, shpkp3-1 and shpkp3-2) and both puromycin and blasticidin for shpkp3-2 derived vector control and double knockdown clones. In three weeks, the total number of colonies formed on the soft agar plate was counted. Three independent experiments were performed in triplicates for each clone.

### Tumor formation in nude mice

BALB/c Nude mice (CAnN.Cg-*Foxn1nu*/Crl) of 6–8 weeks old, provided by the ACTREC animal house facility, was used for the study. 1 x 10^6^ cells of the HCT116 based shpkp3-2 derived vector control and double knockdown clones were resuspended in 100μl of PBS and injected sub-cutaneously in the dorsal flank of mice. Six mice were injected for each clone. Tumor formation was monitored at intervals of 2 to 3 days and tumor size was calculated weekly for 5 weeks using the formula (0.5x LV^2^) where L is the largest dimension and V its perpendicular dimension [24]. The maximum tumor volume of 1045.421 mm^3^ was obtained 5th week post-injection for a mouse injected with shpkp3-2+vec.No surgical procedure was involved in the present study and therefore no anesthetic or analgesic was employed during these experiments. During injections, the animals were handled by trained, certified animal technicians and were injected by the in-house veterinarian with minimum distress to animals. Mice were sacrificed 5 weeks post injection. Animals were euthanized as per in-house Standard Operating Procedure (SOP) approved by the attending veterinarian (AV) of the ACTREC animal house facility. Carbon dioxide (CO_2_), an inhalant euthanasia agent recommended by the Committee for the Purpose of Control and Supervision of the Experiments on Animals (CPCSEA), Government of India, was used for euthanasia of mice. Euthanasia was performed under the supervision of the attending veterinarian and according to the American Veterinary Medical Association (AVMA) guidelines for the euthanasia of animals (2013 Edition). Briefly, a compressed CO_2_ cylinder was used as a source for carbon dioxide to control the inflow of gas, which was connected to a euthanasia chamber. The mice were kept in the chamber and an optimal flow rate was maintained to fill 20% of the chamber volume. After keeping the mice in the chamber, the CO_2_ cylinder supply valve was turned on to deliver the gas in the chamber so that animals were exposed to the gas slowly and steadily. After sufficient exposure like for 2 to 3 minutes, mice showed cessation of respiration and heart beats. The chamber was not prefilled with CO_2_ and was vented out post sacrifice and before the next animal was introduced into the chamber. Thereafter, the mice were removed from the chamber and a cervical dislocation performed to ensure that the mice were dead.

### Ethics statement

Animals were maintained in the ACTREC animal house facility following the national guidelines mentioned by the Committee for the Purpose of Control and Supervision of the Experiments on Animals (CPCSEA), Ministry of Environment and Forest, Government of India. A controlled environment was provided to the animals with a temperature of 22±2°C and relative humidity maintained at 40–70%. A 12 hours day night cycle was maintained (7:00 to 19:00 day and 19:00 to 7:00 night). The animals were given autoclaved balanced diet prepared in house and sterile water. Individually ventilated Cage system (IVC, M/S Citizen, India) was used to house mice used in the experiments. These IVCs were provided with autoclaved corn cob as bedding for the mice. Animal euthanasia was done under the guidelines of AVMA as mentioned above using CPCSEA recommended euthanizing agent, carbon dioxide. The Institutional Animal Ethics Committee (IAEC) of the Advanced Centre for Treatment Research and Education in Cancer (ACTREC) approved all the protocols used in this report. The project number for the study is 16/2008 and was approved in November 2008.

## Results

### PKP3 loss leads to alterations in the transcriptome of multiple cell types

Previous studies from our laboratory have demonstrated that loss of PKP3 in HCT116, HaCaT and FBM cells leads to an increase in transformation in vitro and increased tumor formation and metastasis in vivo [24]. To identify mechanisms downstream of PKP3 loss leading to tumor progression and metastasis, a gene expression analysis was performed to compare the transcriptome of the vector control (vec) and a PKP3 knockdown clone (shpkp3-2) derived from FBM cells. The mRNA purified from the FBM derived vector control (vec) PKP3 knockdown clones (shpkp3-2) was used to perform a single hybrid gene expression microarray using the 8x60K format. The data from these experiments has been uploaded to the NCBI GEO database (Accession no. GSE61512). The microarray was performed in duplicate and the analysis used the average values from the two sets of microarray data. The expression of 427 genes was up-regulated and 428 genes down-regulated in the PKP3 knockdown clone as compared to the vector control. An alteration in the expression of many of genes previously reported to be in pathways regulating inflammation were identified in the microarray (S1A Fig and S2 Table), which is consistent with an increase in inflammation being a hallmark of many tumors (reviewed in [37]). A microarray analysis was also to compare the transcriptome of the vector control (vec) and a PKP3 knockdown clone (shpkp3-2) derived from the HCT116 cells. The data from these experiments has been uploaded to the NCBI GEO database (Accession no. GSE64580). The results from the microarray analysis along with the functional classification of the altered genes upon PKP3 loss are shown in S1A Fig and S3 Table. As PKP3 knockdown leads to an increase in cellular transformation in three different cell lines (HaCaT, HCT116 and FBM), the data obtained from the microarray analysis was used as a reference to identify genes whose expression was altered upon loss of PKP3.

**Fig 1:**
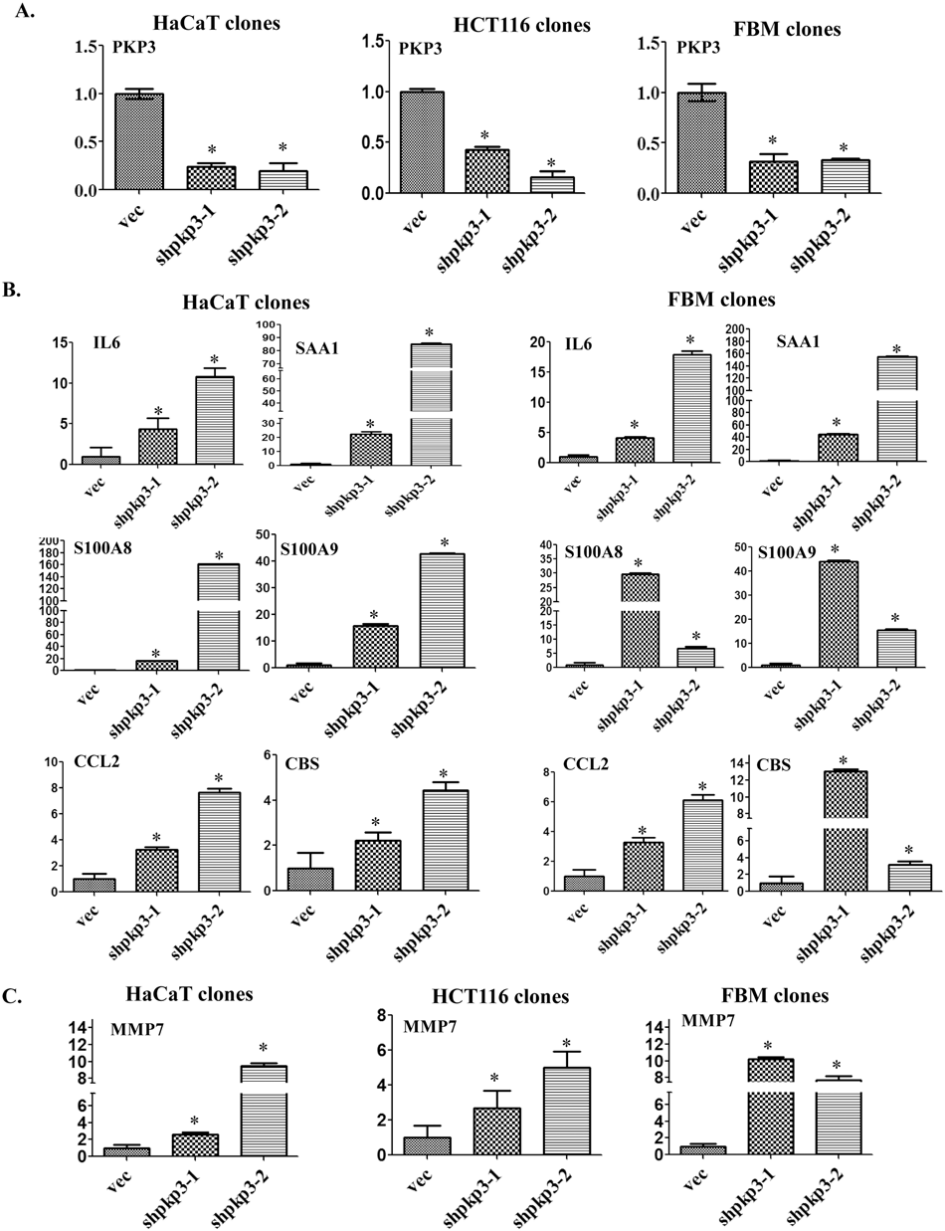
PKP3 loss leads to the generation of an inflammation associated signature in cell lines derived from stratified epithelia. The Y-axis in all panels reflects the fold change in transcription, which is calculated as described in the Materials and Methods. The X-axis indicates the clone name. mRNA was prepared from the vector controls (vec) or PKP3 knockdown clones (sh-pkp3-1 and shpkp3-2) derived from either HCT116, FBM or HaCaT cells as indicated. **(A)** Real time PCR assays were performed using oligonucleotides specific to PKP3 and GAPDH. Relative expression of PKP3 in the HCT116, HaCaT and FBM derived PKP3 knockdown clones (shpkp3-1 and shpkp3-2) was compared to the respective vector control clones (vec). Expression of GAPDH has been used for normalization. **(B)** Real time PCRs were performed using oligonucleotides specific for IL6, SAA1, S100A8, S100A9, CCL2, CBS and GAPDH, in HaCaT and FBM derived PKP3 knockdown clones and the respective vector controls. Expression of GAPDH has been used for normalization. **(C)** Real time PCR was performed using oligonucleotides specific to MMP7 and GAPDH, with cDNA obtained from the vector control and PKP3 knockdown clones derived from the three cell types under study. Expression of GAPDH has been used for normalization. The standard errors are plotted and student’s t test was performed (* indicates a p value <0.01).

In confirmation of previously reported results [24, 30], a real time PCR analysis demonstrated that the mRNA levels of PKP3 was reduced in the PKP3 knockdown clones as compared to the vector controls (Fig 1A). The expression of genes which were found to be up-regulated or down-regulated more than two-fold in the FBM derived PKP3 knockdown clone was assessed using real time PCR to validate the results of the microarray and to analyze if the same genes were altered upon PKP3 knockdown in the HCT116 and HaCaT cell lines. A change in expression of two-fold either way was considered significant. A set of genes which included inflammation associated genes such as Interleukin 6 (IL6), Serum amyloid A1 (SAA1), Chemokine (C-C Motif) Ligand 2 (CCL2), S100A8, S100A9 and CBS, were up-regulated in HaCaT and FBM derived PKP3 knockdown clones (Fig 1B). None of these genes were expressed in HCT116 cells (data not shown). The expression of Epiplakin (EPPK1), Rho Guanine Nucleotide Exchange Factor (ARHGEF5), Matrix metalloprotease 9 (MMP9), MOB kinase activator 3B (MOBKL2b) and N terminal deleted isoform of TAp63 (ΔNp63) was found to be up-regulated while expression of Nuclear receptor subfamily-2 group-F member-1 (NR2F1) and Insulin like growth factor binding protein 3 (IGFBP3) was found to be down-regulated only in the FBM derived PKP3 knockdown clones while they their expression was not altered in the HaCaT and HCT116 derived PKP3 knockdown clones (S1B Fig). The expressions of all the genes were normalized using the expression of GAPDH as a control as described in the Materials and Methods. These results suggested that PKP3 loss leads to varying alterations in the transcriptome in the three cell types studied and that PKP3 loss leads to the generation of an inflammation associated signature in cell lines derived from stratified epithelia, which is consistent with the observation that inflammation is observed in the epidermis of mice lacking PKP3 [29].

### MMP7 is required for transformation upon loss of PKP3

**Fig 2:**
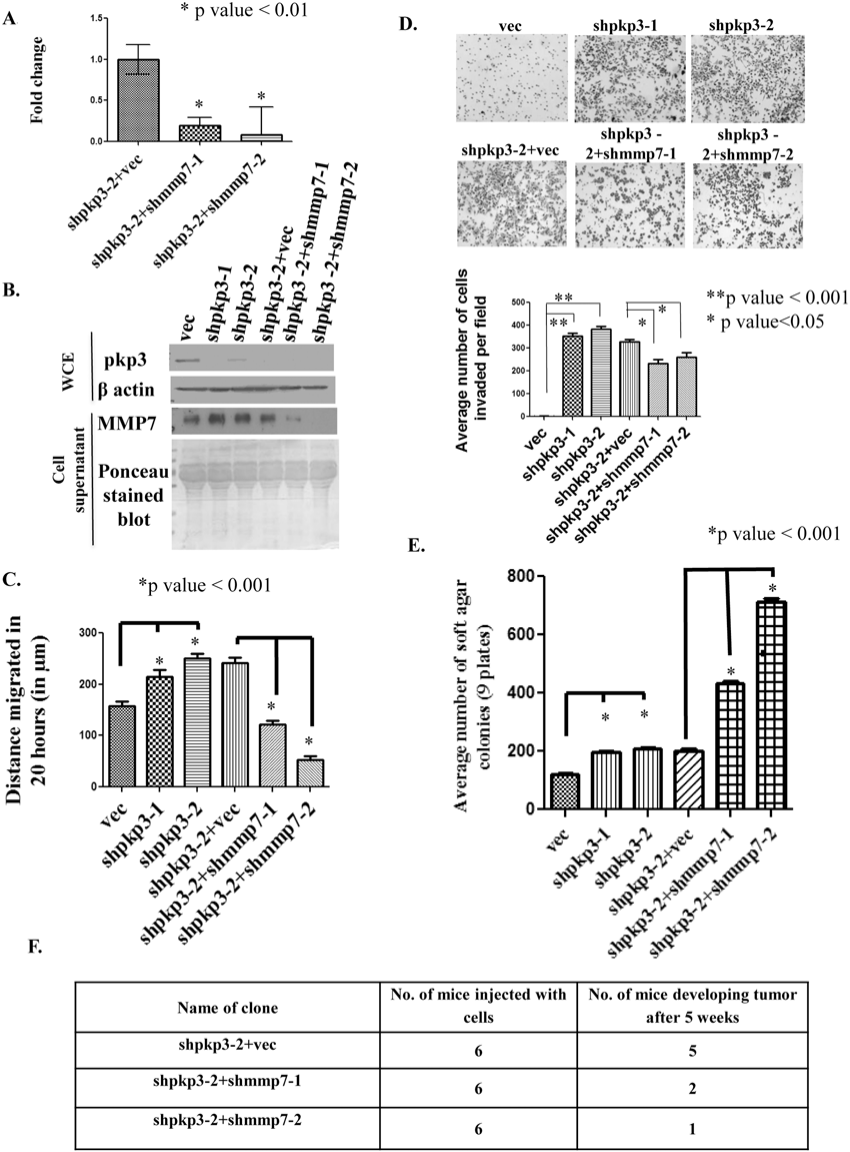
Loss of MMP7 leads to a decrease in transformation in cells lacking PKP3. **(A)** mRNA prepared from HCT116 derived PKP3 knockdown cells transfected with the vector control (shpkp3-2 + vec) or the MMP7 knockdown construct (shpkp3-2 + shMMP7-1 and shpkp3-2 + MMP7-2) was used as a substrate for reverse transcriptase followed by real time PCR reactions using oligonucleotides specific for MMP7. All expression was normalized to the levels of GAPDH. The fold change is graphed on the Y-axis and the clone name is on the X-axis. Note that MMP7 levels are lowered in the double knockdown clones as compared to the vector control. The standard errors are plotted and student’s t test was performed (* indicates a p value <0.01). **(B)** 75μg of a whole cell extract (WCE) was resolved on 12% SDS PAGE gels followed by Western blotting with antibodies specific to PKP3. Note that PKP3 levels are lower in clones with a PKP3 knockdown. Western blots for β actin served as a loading control (upper panels). 100μg of acetone precipitated cell supernatants were resolved on 12% SDS PAGE gels followed by Western blotting with antibodies specific to MMP7. Note that MMP7 levels are higher in supernatants prepared from the PKP3 knockdown cells as compared to the vector controls and the levels are lower in the double knockdown clones. The same blot was stained with Ponceau stain to demonstrate equal loading of proteins (lower panels). **(C)** Scratch wound healing assays were performed on the HCT116 derived vector control (vec), PKP3 knockdown clones (shpkp3-1 and shpkp3-2), shpkp3-2 derived vector control clone (shpkp3-2+vec) and shpkp3-2 derived MMP7 knockdown clones (shpkp3-2+shMMP7-1 and shpkp3-2+shMMP7-2) as described. **(D)** Matrigel invasion assays were performed in Boyden’s chambers for HCT116 derived vector control cells, PKP3 knockdown clones and the double knockdown clones. The number of cells observed in ten random fields of the membrane for each clone was determined as described in Materials and Methods, representative images of for each clone are shown. The mean and standard deviation of three independent experiments are plotted. Note that loss of PKP3 leads to an increase in invasion as compared to the vector control and this phenotype is reversed in the double knockdown clones. **(E)** Soft agar colony formation assay was performed with the HCT116 derived vector control and pkp3 knockdown clones, the shpkp3-2 derived double knockdown clones and the shpkp3-2 derived vector control clone. The mean and standard deviation of three independent experiments is plotted. **(F)** 1x10^6^ cells of the shpkp3-2 derived vector control or double knockdown clones were injected sub-cutaneously into nude mice and allowed to develop tumors. The table shows the number of mice injected with the respective clones and the number of mice among them which were able to develop tumors. Wherever indicated the p value was calculated using a student’s t-test.

The mRNA levels of the Matrix metalloprotease7 (MMP7) gene were found to be up-regulated upon PKP3 loss in all three cell types tested (Fig 1C). To determine whether the increase in MMP7 levels is required for tumor formation upon PKP3 loss, double knockdown clones were generated where MMP7 was knocked down in the PKP3 knockdown clone shpkp3-2 using vector driven RNA interference. Two double knockdown clones were obtained, shpkp3-2+shMMP7-1 and shpkp3-2+shMMP7-2. The vector control clone (shpkp3-2+vec) was generated by transfection of the empty vector in the shpkp3-2 clone. The expression of MMP7 in the vector control and the double knockdown clones was validated by real time PCR (Fig 2a). Since MMP7 is a secreted protein [6, 38, 39], cells were grown in the absence of serum and the supernatant collected for the indicated cell types. The proteins in the supernatant were precipitated using acetone as described in the Materials and Methods and a Western blot performed for MMP7. The cells were lysed and harvested for Western blots performed for PKP3 and actin as described [30]. PKP3 levels were reduced in both the single and double knockdown clones (Fig 2B). A Western blot for β actin served as a loading control. PKP3 knockdown led to an increase in the expression of MMP7 while knockdown of MMP7 causes a decrease in the expression of MMP7 as expected (Fig 2B). The blot was stained with ponceau-S to show equal protein loading for the cell supernatants.

PKP3 loss leads to an increase in cell migration as assayed by scratch wound healing assays [24]. To determine if MMP7 loss can reverse the increase in cell migration observed upon PKP3 loss, scratch wound healing assays were performed. As reported earlier, pkp3 loss increased in-vitro cell migration of HCT116 cells, while MMP7 loss in the pkp3 knockdown clones decreased in-vitro cell migration in PKP3 knockdown cells (Fig 2C and S1C Fig). PKP3 loss leads to an increase in metastasis and this is often associated with an increase in cell invasion. To determine if PKP3 loss leads to increased invasion, matrigel invasion assays were performed as described [35, 36]. Loss of PKP3 leads to an increase in invasion as shown in Fig 2D. The increase in invasion observed upon PKP3 loss was reversed when MMP7 expression was inhibited (Fig 2D). These results suggested that MMP7 is required for the increased migration and invasion observed upon PKP3 knockdown in HCT116 cells.

PKP3 loss leads to an increase in anchorage independent growth and an increase in tumor formation and metastasis in vivo [24]. To determine the effect of MMP7 loss on anchorage independent growth soft agar assays were performed on the vector control and double knockdown clones. As reported earlier, pkp3 loss increased anchorage independent growth, but surprisingly, the double knockdown clones formed more colonies in soft-agar than the vector control clones (Fig 2E). Since this result was in contrast to the results obtained in the migration and invasion assays, we determined whether MMP7 loss leads to an alteration in tumor formation in vivo. It has been reported earlier that HCT116 derived pkp3 knockdown clones form larger tumors and show increased metastasis in vivo [24]. The vector control, shpkp3-2+vec and the double knockdown clones were injected subcutaneously in nude mice as previously described [24, 30]. The mice were observed for 5 weeks and tumor size measured at regular intervals. Five of the six mice injected with the vector control developed large tumors Fig 2F and S2A Fig). In contrast, only two out of six mice injected with shpkp3-2+shMMP7-1 and one of the six mice injected with shpkp3-2+shMMP7-2 were able to develop tumors at the site of injection and the tumors formed were much smaller in size than those formed in mice injected with the vector control clone (shpkp3-2+vec)(Fig 2F and S2A Fig). The average volume of the tumor formed in the mice was analyzed using the formula mentioned in Materials and Methods (data not shown).

### PRL3 regulates MMP7 expression in the HCT116 derived PKP3 knockdown clones

**Fig 3:**
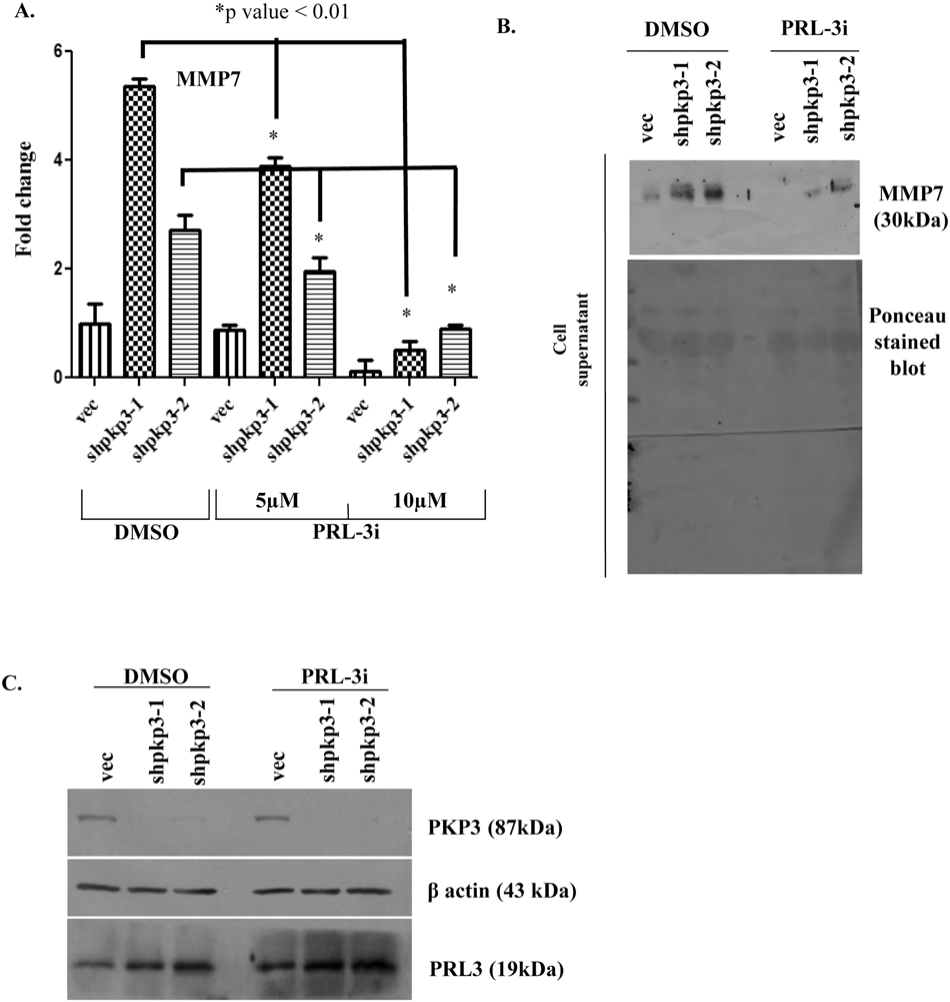
MMP7 expression decreases upon inhibition of PRL3 activity. **(A)** The HCT116 derived PKP3 knockdown clones (shpkp3-1 and shpkp3-2) or the vector control (vec) were treated with either the vehicle control (DMSO) or 5 or 10 μM PRL-3 inhibitor-1(PRL-3i) for 24 hours. The mRNA prepared from the treated cells was used as a substrate for reverse transcriptase followed by real time PCR reactions using oligonucleotides specific for MMP7. All expression was normalized to the levels of GAPDH. The fold change is graphed on the Y-axis and the clone name is on the X-axis. The standard errors are plotted and student’s t test was performed. Note that MMP7 levels are lowered upon treatment with PRL-3 inhibitor. **(B)** The HCT116 derived vector control (vec) and PKP3 knockdown clones (shpkp3-1 and shpkp3-2) were treated with either DMSO or 10 μM PRL3 inhibitor-1(PRL3i) for 24 hours or 48 hours. The cell supernatants were collected and a100μg of acetone precipitated protein was resolved on 12% SDS PAGE gels followed by Western blotting with antibodies to MMP7. The same blot was stained with Ponceau stain to indicate equal loading. **(C)** The whole cell lysates of HCT116 derived vector control and plakophilin3 knockdown clones treated with DMSO or PRL3i for 24 hours were resolved on 12% poly-acrylamide gel. This was followed by Western blotting with antibodies to PKP3, β actin and PRL3. The molecular weights of these proteins are indicated in brackets.

Previous data from our laboratory has demonstrated that an elevation in the levels of Keratin 8 is observed in the HCT116 derived PKP3 knockdown clones and that this increase is due to an increase in the levels of the phosphatase of regenerating liver 3 (PRL-3) and inhibition of PRL-3 activity using a chemical inhibitor resulted in a decrease in cell migration [30]. An inhibition of PRL-3 activity using a chemical inhibitor of PRL-3 resulted in a decrease in cell migration in the HCT116 derived PKP3 knockdown clones [30]. A previous report demonstrated that PRL3 activity regulates MMP7 expression via the PI3K/AKT and ERK signaling pathway in the colon cancer derived cell line DLD1 [40]. Therefore, we hypothesized that the increase in MMP7 levels observed upon PKP3 knockdown was due to the increase in PRL-3 levels. To test this hypothesis the HCT116 derived vector control and PKP3 knockdown clones were treated with 5 or 10μM of the PRL-3 inhibitor for 24 hours. A real time PCR analysis demonstrated that the levels of MMP7 mRNA in the PKP3 knockdown clones were decreased upon treatment with the PRL-3 inhibitor in a concentration dependent manner (Fig 3A). The mRNA levels of Lipocalin2 (LCN2) which is increased upon PKP3 knockdown in HCT116 cells, did not decrease upon treatment with the PRL-3 inhibitor (S2B Fig) suggesting that PRL3 activity does not regulate the expression of other gene products such as LCN2 in these cells. A Western blot analysis demonstrated that the levels of MMP7 protein were also decreased upon treatment with the inhibitor at 24 hours in HCT116 cells (Fig 3B), but not in the HaCaT derived PKP3 knockdown clones (S2C Fig). To determine whether PRL3 inhibitor treatment (which inhibits the activity of PRL3) resulted in a change in PRL-3 protein levels, a Western blot analysis was performed. It was observed that PRL-3 levels were higher in PKP3 knockdown clones as reported earlier [30], but no change in the levels of PRL-3 was observed upon treatment with the PRL-3 inhibitor (Fig 3C). A Western blot for actin served as a loading control. These results suggest that the increase in PRL-3 levels observed upon PKP3 loss is required for the increase in MMP7 mRNA and protein levels observed in HCT116 cells.

## Discussion

The results in this report demonstrate that loss of PKP3 leads to an increase in the levels of MMP7 in three independently derived cell lines of different origins. The increase in MMP7 levels is required for cell migration and invasion in vitro and tumor formation in nude mice as loss of MMP7 in the PKP3 knockdown cells results in a reversal of these phenotypes. The increase in MMP7 mRNA is dependent on the expression of PRL-3, whose levels are elevated upon PKP3 loss [30]. Thus, the results in this paper suggest that the increased tumor formation observed upon PKP3 loss requires the expression of MMP7.

### MMP7 mediates cell invasion and tumor formation upon PKP3 loss

MMP7 and membrane type-1 matrix metalloprotease (MT1-MMP) are metalloproteases which are exclusively produced by the epithelial cells of the colon while the rest of these matrix-metalloproteases such as MMP9, MMP11 (stromelysin 3) are produced by the stroma [3, 41–43]. MMP7 levels are increased in colon cancer tissues [4–6] and in the serum of colon cancer patients [7]. A study by Witty et.al., demonstrated that MMP7 knockdown clones derived from the colon cancer cell line SW620 do not form tumors as efficiently as the corresponding vector controls in orthotopic xenografts of colon cancer [18]. Further, studies by Wilson et. al., [16] and Guillen-Ahlers et.al. [17] demonstrated that loss of MMP7 in the intestine of Apc^Min^ mice leads to a decrease in tumor incidence. Our work has demonstrated that PKP3 loss leads to an increase in migration, invasion, tumor formation and metastasis and that these functions are dependent upon the increase in MMP7 expression upon PKP3 knockdown. To determine if a correlation exists between MMP7 and PKP3 levels across colon cancers at the transcript level, we analyzed the data sets in the Oncomine database (www.oncomine.org). When PKP3 levels in normal colon samples were compared with that in colon cancer, the levels were found to be unchanged, while MMP7 levels were higher in colon cancer samples. None of the databases present had actually validated the PKP3 levels using real time PCR or reverse transcriptase PCR. To determine if a correlation exists between MMP7 and PKP3 levels across colon cancers at the protein level, we examined the data deposited in the human protein atlas database (http://www.proteinatlas.org/). It was observed that a thorough immuno-histochemical analysis of PKP3 and MMP7 levels in a large dataset of colorectal cancer patients has not been performed to determine if any correlation exists between PKP3 and MMP7 levels. Surprisingly, MMP7 loss leads to an increase in colony formation in soft agar but a decrease in tumor formation in nude mice. These results suggest that the ability of MMP7 to induce the degradation of the extra-cellular matrix in vivo maybe essential for the ability of the PKP3 knockdown cells to form a tumor. Taken together these results suggest that the increase in MMP7 levels observed upon PKP3 loss is required for increased tumorigenesis in cells derived from the colon.

### MMP7 over-expression upon PKP3 is regulated by PRL3

PRL-3 levels are increased in colorectal cancers [44] and PRL-3 expression leads to metastasis in tumors derived from the colon [45]. Lee et.al. [40] demonstrated that PRL3 increases cell migration and invasion by up-regulating expression of MMP7 in colorectal cancer cell line DLD-1, via the PI3K/AKT and ERK signaling pathway. Earlier reports from this laboratory have demonstrated that an increase in K8 levels upon PKP3 knockdown in HCT116 cells is dependent on increased levels of PRL-3 and that the inhibition of PRL-3 expression using shRNA constructs or the inhibition of PRL-3 activity using a specific inhibitor results in a reversal of the observed phenotype [30]. Similarly, the results in this paper demonstrate that inhibition of PRL-3 activity in the PKP3 knockdown clones results in a decrease in MMP7 levels. No increase in the levels of PRL-3 mRNA or stability was observed in HCT116 derived PKP3 knockdown cells [30] suggesting that the increase in PRL-3 protein levels occurred post-transcriptionally. One possible explanation for this observation is that PRL3 translation maybe increased upon PKP3 loss. Hofmann, et.al have demonstrated that PKP3 localizes to stress granules (sites of stalled mRNA-protein complexes) and forms a complex with RNA binding proteins like PolyA binding protein Cytoplasmic 1 (PABPC1), Fragile X Mental Retardation, Autosomal Homolog 1 (FXR1), and GTPase Activating Protein (SH3 Domain) Binding Protein 1 (G3BP) [46, 47]. Thus, PKP3 may regulate the translation of PRL-3 mRNA. Consistent with these observations, plakophilin1 has been reported to regulate translation initiation by directly binding to eIF4A (eukaryotic Initiation factor 4A) and promoting its activity [48].

### PKP3 knockdown leads to differential alterations in transcriptome of three different cell types

PKP3 knockout mice show symptoms of severe itching, intercellular edema, neutrophil infiltration, epidermal hyperplasia, hair loss and muscle wasting [29]. Because of this dermatitis associated muscle wasting, the PKP3 null mice are smaller in size than the wild type mice and are very similar to the mouse model for atopic dermatitis, a severe form of skin inflammation. A previous gene expression analysis demonstrated that conditional deletion of the desmosomal protein Perp in stratified epithelia of mice led to up-regulation of inflammatory associated genes like S100A9 and the Chemokine (C-C motif) ligand 20 (CCL20)[49]. Another transcriptomic analysis performed by Jheon et.al.[50], demonstrated that Perp loss in the enamel of mice leads to alterations in gene expression. Consistent with these observations, in this study we report that loss of PKP3 in cell lines derived from the HaCaT and FBM cell lines lead to the over-expression of multiple genes associated with inflammation (Fig 1B). These results provide a possible explanation for the skin inflammation phenotype obtained in PKP3 knockout mice [29] and maybe dependent on the loss of desmosome function observed upon PKP3 loss [23, 24]. To our surprise MMP7 levels did not decrease in the HaCaT cells upon treatment with the PRL-3 inhibitor suggesting that the increase in MMP7 in HaCaT cells occurs via mechanisms distinct from those observed in HCT116 cells and is consistent with our observations that PKP3 loss leads to cell type specific alterations in the transcriptome. The relevance of other alterations in gene expression that are specific to the FBM line is not clear. They may relate to the role of PKP3 in regulating desmosome formation in the oral cavity or these changes might have a role to play in the increased migration and cellular transformation observed in these cells upon PKP3 loss [24]. Thus, PKP3 loss can lead to both cell type dependent and cell type independent alterations in gene expression.

The results reported here lead to the generation of the following model. PKP3 loss leads to an increase in PRL-3 translation leading to an increase in K8 levels [30] or an increase in MMP7 mRNA levels (this report). Both K8 and MMP7 are independently required for the increase in migration and transformation observed upon PKP3 loss suggesting that either one can serve as a potential drug target in metastatic colon cancer. The role of PKP3 in regulating the increase in PRL-3 protein levels remains to be determined and should be a focus for future investigation.

## Supporting Information

S1 Table List of oligonucleotides used in the study. (DOCX)

S2 Table Alterations in gene expression upon PKP3 knockdown in FBM cells and functional classification of the altered genes. Compiled data with complete dataset, differentially expressed genes and functional classification of altered genes observed upon PKP3 knockdown in FBM cell line.
(XLSX)

S3 Table Alterations in gene expression upon PKP3 knockdown in HCT116 cells and functional classification of the altered genes. Compiled data with complete dataset, differentially expressed genes and functional classification of altered genes observed upon PKP3 knockdown in HCT116 cell line.
(XLSX)

S1 Fig S1A Fig, Functional classification of genes whose expression is altered >=2 folds in FBM derived clones and >=1.5 folds in HCT116 derived clones upon PKP3 knockdown. The mRNA expression profile of the FBM derived vector control (vec) was compared with that of the PKP3 knockdown clone (shpkp3-2). Similarly, mRNA expression profile of the HCT116 derived vector control (vec) was compared with that of the PKP3 knockdown clone (shpkp3-2). Differentially expressed genes which show more than two fold (for FBM) and more than 1.5 folds up-regulation or down-regulation in the PKP3 knockdown clones compared to the vector control clone were selected. The pie charts show functional classification of genes obtained from the microarray data analysis using the PANTHER Classification tool (http://www.pantherdb.org/about.jsp). S1B Fig, Cell type specific alterations in FBM derived PKP3 knockdown clones. Reverse transcriptase PCRs were performed using oligonucleotides specific for SAA4, EPPK1, MOBKL2b, MMP9, NR2F1, ΔNp63, IGFBP3, ARHGEF5 and GAPDH, in HaCaT, HCT116 and FBM derived PKP3 knockdown clones and the respective vector controls. Expression of GAPDH has been used for normalization. The PCR product size has been indicated in base pairs. S1C Fig, MMP7 loss leads to a decrease in migration. Phase contrast images of wound healing at 0 hours (start) and 20 hours (end of experiment) have been shown.
(TIF)

S2 Fig S2A Fig, Tumor formation is inhibited in the PKP3 knockdown cells upon MMP7 knockdown. 10^6^ cells of the vector control or the double knockdown clones were injected sub-cutaneously into nude mice and allowed to develop tumors. Representative images of mice have been shown. S2B Fig, LCN2 expression is not altered upon inhibition of PRL-3. The HCT116 derived PKP3 knockdown clones (shpkp3-1 and shpkp3-2) or the vector control (vec) were treated with either the vehicle control (DMSO) or 10 μM PRL-3 inhibitor-1 (PRL-3i) for 24 hours. The mRNA prepared from the treated cells was used as a substrate for reverse transcriptase followed by real time PCR reactions using oligonucleotides specific for LCN2. All expression was normalized to the levels of GAPDH. The fold change is graphed on the Y-axis and the clone name is on the X-axis. The standard errors are plotted and student’s t test was performed (* indicates a p value <0.01). Note that LCN2 levels are increased upon treatment with PRL-3 inhibitor. S2C Fig, MMP7 expression does not change upon inhibition of PRL3 activity in HaCaT derived clones. The HaCaT derived PKP3 knockdown clones (shpkp3-1 and shpkp3-2) or the vector control (vec) were treated with either the vehicle control (DMSO) or 10 μM PRL-3 inhibitor-1(PRL-3i) for 24 hours. The cell supernatants were collected and a100μg of acetone precipitated protein was resolved on 12% SDS PAGE gels followed by Western blotting with antibodies to MMP7. The same blot was stained with Ponceau to indicate equal loading.
(TIF)
