# Supplementary material for: MMP7 Is Required to Mediate Cell Invasion and Tumor Formation upon Plakophilin3 Loss
Source: PLoS One. 2015 Apr 13;10(4):e0123979. doi: 10.1371/journal.pone.0123979 (PMC4395386; doi:10.1371/journal.pone.0123979)
Supplement: S1 Table — (DOCX) [file pone.0123979.s001.docx]

**S5 Table. List of oligonucleotides used in the study.**

| **Name of gene** | **Sequence (5’ to 3’)** |
| --- | --- |
| GAPDH Forward | TGCACCACCAACTGCTTAGC |
| GAPDH Reverse | GGCATGGACTGTGGTCATGAG |
| PKP3 Forward | TGATGAGCTTCGCAAAAATG |
| PKP3 Reverse | CTGAGAGGCTGAGCTGAGGT |
| MMP7 Forward | ACAGTGGGAACAGGCTCAGGACT |
| MMP7 Reverse | TCTGGCACTCCACATCTGGGC |
| IL6 Forward | GCCACTCACCTCTTCAGAACGAATTG |
| IL6 Reverse | CCAGATTGGAAGCATCCATC |
| SAA1 Forward | CTGCAGAAGTGATCAGCG |
| SAA1 Reverse | ATTGTGTACCCTCTCCCC |
| S100A8 Forward | CAGTATATCAGGAAAAAGGGTGC |
| S100A8 Reverse | GCCACGCCCATCTTTATCA |
| S100A9 Forward | TCATCAACACCTTCCACCAA |
| S100A9 Reverse | TTTGTGTCCAGGTCCTCCAT |
| CCL2 Forward | GTCTCTGCCGCCCTTCTGTGC |
| CCL2 Reverse | AACAGCAGGTGACTGGGGCAT |
| CBS Forward | AAGTTGGCAAAGTCATCTACA |
| CBS Reverse | CAGCAAGTCAATGGCGGTG |
| SAA4 Forward | GTTCGTTTTTCAAGGAGGCT |
| SAA4 Reverse | TCCCTGAAGATAGACCCTGG |
| EPPK1 Forward | AGCTGGTGAGGATGTATAGAACACAC |
| EPPK1 Reverse | TGTTTGTTGCTGGTTTCCTGC |
| ARHGEF5 Forward | GCCAACAAGCACAAGGGCTGGA |
| ARHGEF5 Reverse | AGCTGGGAGGAGTTGATGAGTTTGG |
| MMP9 Forward | GGGGGAAGATGCTGCTGTT |
| MMP9 Reverse | GGCTTTCTCTCGGTACTGGA |
| MOBKL2B Forward | GTTCTGCACCGAGCGGACCTG |
| MOBKL2B Reverse | GGGAACACCCACGCATGTTGGA |
| ΔNp63 Forward | CTGGAAAACAATGCCCAGAC |
| ΔNp63 Reverse | GGGTGATGGAGAGAGAGCAT |
| NR2F1 Forward | CATCGTGCTGTTCACGTCAGACGCC |
| NR2F1 Reverse | GGGCAGTCGCAGCAGCAGTTT |
| IGFBP3 Forward | TGACGTGCGCACTGAGCGAG |
| IGFBP3 Reverse | GCTCACCTGGAGCTGGCGG |
| LCN2 Forward | CCTCTACGGGAGAACCAAGGAGC |
| LCN2 Reverse | ACCTGTGCACTCAGCCGTCG |
| shRNA1 MMP7 Forward | CCGGAACAGGCTCAGGACTATCTCACTCGAGTGAGATAGTCCTGAGCCTGTTTTTTTG |
| shRNA1 MMP7 Reverse | AATTCAAAAAAACAGGCTCAGGACTATCTCACTCGAGTGAGATAGTCCTGAGCCTGTT |
