# Supplementary figures and images for: MMP7 Is Required to Mediate Cell Invasion and Tumor Formation upon Plakophilin3 Loss
Source: PLoS One. 2015 Apr 13;10(4):e0123979. doi: 10.1371/journal.pone.0123979 (PMC4395386; doi:10.1371/journal.pone.0123979)

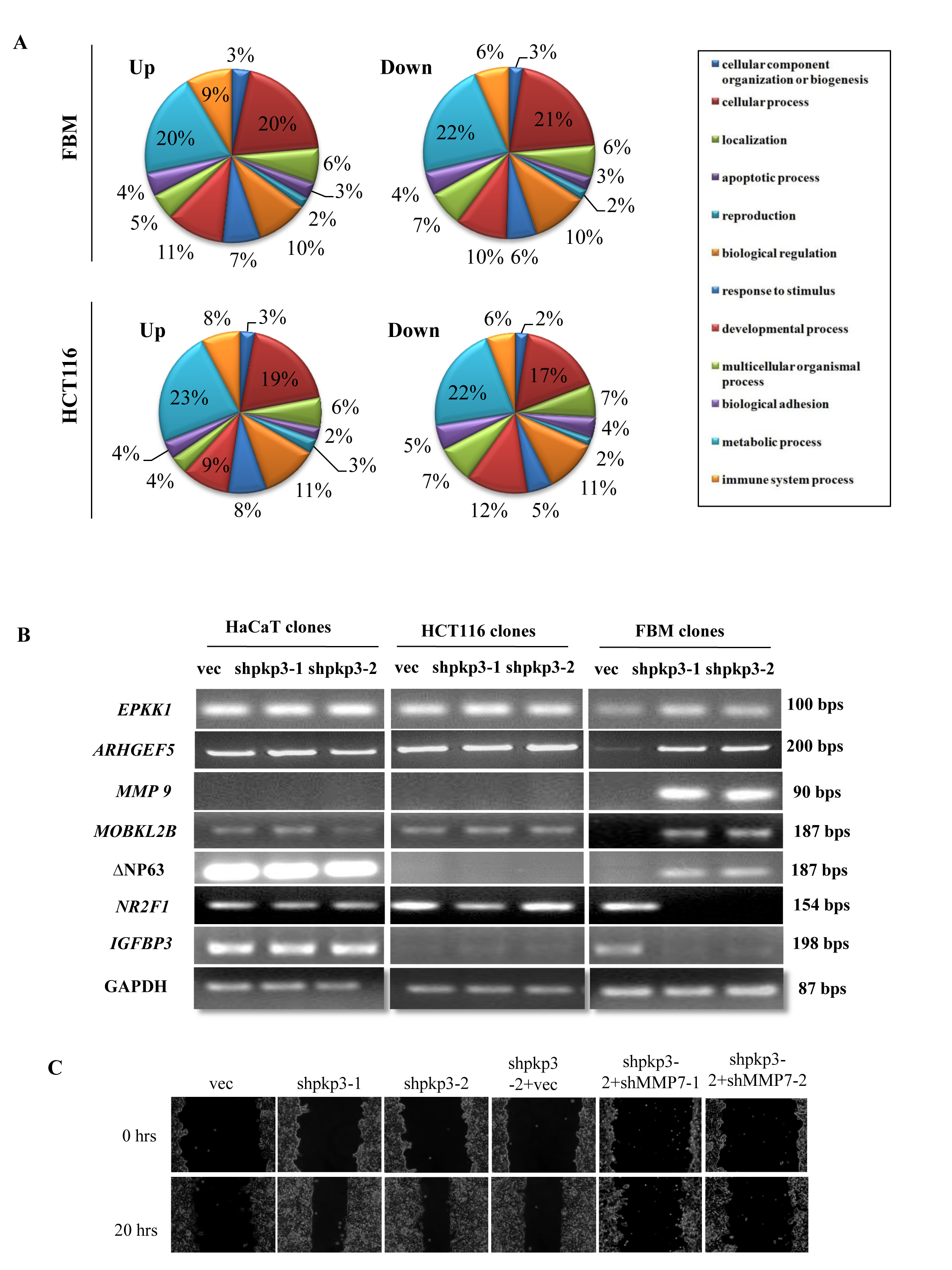

Supplement: S1 Fig — S1A Fig, Functional classification of genes whose expression is altered >=2 folds in FBM derived clones and >=1.5 folds in HCT116 derived clones upon PKP3 knockdown. The mRNA expression profile of the FBM derived vector control (vec) was compared with that of the PKP3 knockdown clone (shpkp3-2). Similarly, mRNA expression profile of the HCT116 derived vector control (vec) was compared with that of the PKP3 knockdown clone (shpkp3-2). Differentially expressed genes which show more than two fold (for FBM) and more than 1.5 folds up-regulation or down-regulation in the PKP3 knockdown clones compared to the vector control clone were selected. The pie charts show functional classification of genes obtained from the microarray data analysis using the PANTHER Classification tool (http://www.pantherdb.org/about.jsp). S1B Fig, Cell type specific alterations in FBM derived PKP3 knockdown clones. Reverse transcriptase PCRs were performed using oligonucleotides specific for SAA4, EPPK1, MOBKL2b, MMP9, NR2F1, ΔNp63, IGFBP3, ARHGEF5 and GAPDH, in HaCaT, HCT116 and FBM derived PKP3 knockdown clones and the respective vector controls. Expression of GAPDH has been used for normalization. The PCR product size has been indicated in base pairs. S1C Fig, MMP7 loss leads to a decrease in migration. Phase contrast images of wound healing at 0 hours (start) and 20 hours (end of experiment) have been shown. (TIF) [file pone.0123979.s004.tif]

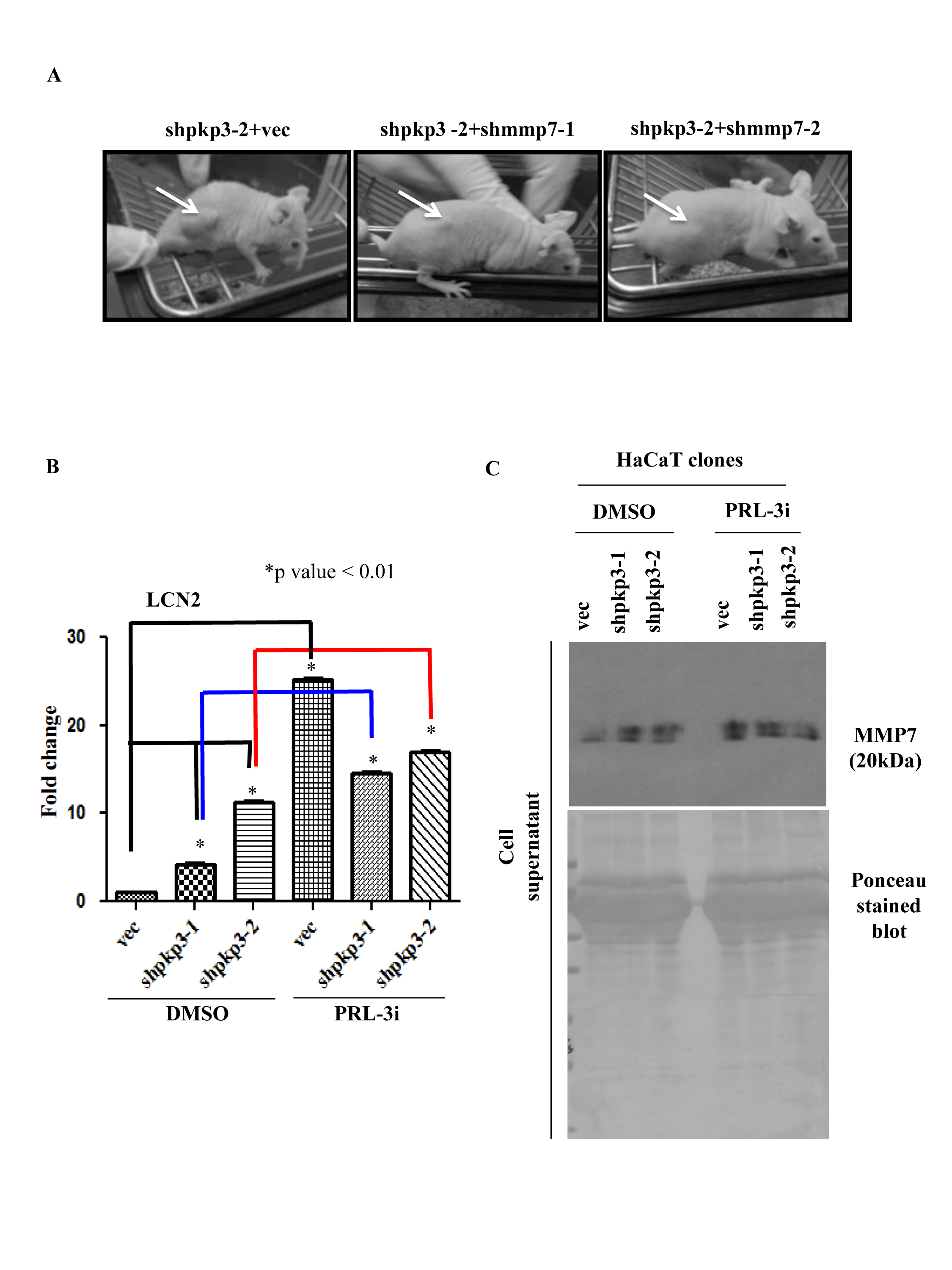

Supplement: S2 Fig — S2A Fig, Tumor formation is inhibited in the PKP3 knockdown cells upon MMP7 knockdown. 106 cells of the vector control or the double knockdown clones were injected sub-cutaneously into nude mice and allowed to develop tumors. Representative images of mice have been shown. S2B Fig, LCN2 expression is not altered upon inhibition of PRL-3. The HCT116 derived PKP3 knockdown clones (shpkp3-1 and shpkp3-2) or the vector control (vec) were treated with either the vehicle control (DMSO) or 10 μM PRL-3 inhibitor-1 (PRL-3i) for 24 hours. The mRNA prepared from the treated cells was used as a substrate for reverse transcriptase followed by real time PCR reactions using oligonucleotides specific for LCN2. All expression was normalized to the levels of GAPDH. The fold change is graphed on the Y-axis and the clone name is on the X-axis. The standard errors are plotted and student’s t test was performed (* indicates a p value <0.01). Note that LCN2 levels are increased upon treatment with PRL-3 inhibitor. S2C Fig, MMP7 expression does not change upon inhibition of PRL3 activity in HaCaT derived clones. The HaCaT derived PKP3 knockdown clones (shpkp3-1 and shpkp3-2) or the vector control (vec) were treated with either the vehicle control (DMSO) or 10 μM PRL-3 inhibitor-1(PRL-3i) for 24 hours. The cell supernatants were collected and a100μg of acetone precipitated protein was resolved on 12% SDS PAGE gels followed by Western blotting with antibodies to MMP7. The same blot was stained with Ponceau to indicate equal loading. (TIF) [file pone.0123979.s005.tif]
